# Role of dedicated port cleaning devices in laparoscopic surgery

**DOI:** 10.1007/s00464-024-11366-w

**Published:** 2024-11-11

**Authors:** Shinnosuke Nagano, Shota Fujii, Kota Momose, Kotaro Yamashita, Takuro Saito, Koji Tanaka, Kazuyoshi Yamamoto, Tomoki Makino, Tsuyoshi Takahashi, Yukinori Kurokawa, Hidetoshi Eguchi, Yuichiro Doki, Kiyokazu Nakajima

**Affiliations:** 1 Department of Next Generation Endoscopic Intervention (Project ENGINE), Osaka University Graduate School of Medicine, 1-3 Yamadaoka, Suita, Osaka 565-0871 Japan; 2 Department of Gastroenterological Surgery, Osaka University Graduate School of Medicine, Osaka, Japan; 3 SANYO Co., LTD, Osaka, Japan

**Keywords:** Laparoscopic surgery, Port cleaning, Cleaning device

## Abstract

**Background:**

Various techniques have been used to prevent smudge on a laparoscope when inserting through trocars; however, there has been no standardized method. The purpose of this study was to compare the performance of different cleaning techniques with or without using dedicated devices, and to evaluate the features of cleaning devices.

**Methods:**

The smudge was created in the standard 12-mm and 5-mm ports using pseudo-blood, and port cleaning was attempted using 5 different methods: (1) a surgical gauze + surgical forceps, (2) a surgical gauze + laparoscopic forceps, (3) a small laparoscopic gauze + laparoscopic forceps, (4) a cylinder-type cleaner (Endo Wiper; Osaki Medical), and (5) a swab-type cleaner (Port Cleaner; Hakuzo Medical). The “port cleaning rate” was calculated by measuring the absorbance of remained pseudo-blood after single cleaning procedure using UV spectrophotometry. In addition, the port cleaning rate was compared between two dedicated devices after multiple (5 times) cleaning procedures.

**Results:**

The two dedicated devices had a statistically higher cleaning rate for 12-mm port than the methods using surgical gauze (p < 0.05). Regarding the 5-mm port, a swab-type cleaner showed the highest cleaning rate than the gauze method and a cylinder-type cleaner (p < 0.05). After multiple cleaning procedures for 12-mm port, cleaning rate of a swab-type cleaner decreased by an average of 5.4% (p = 0.044), but cleaning rate did not decrease for a cylinder-type cleaner. Regarding the 5-mm port, cleaning rate statistically decreased for both two dedicated devices (p < 0.01).

**Conclusion:**

Higher port cleaning rates were observed in techniques using dedicated devices. A swab-type cleaner had better port cleaning rate in single use, especially for the 5-mm port. A cylinder-type cleaner showed higher durability in cleaning 12-mm port. The features of these dedicated devices should be well understood, and cleaning methods should be selected according to the environment and surgical techniques.

**Supplementary Information:**

The online version contains supplementary material available at 10.1007/s00464-024-11366-w.

In recent years, with the development of laparoscopic surgery, the performance of video imaging as the surgeon’s eye has rapidly advanced [1, 2]. The laparoscopic imaging is crucial as it provides a better anatomical understanding and is one of the factors influencing quality of surgery. On the other hand, no matter how good imaging devices are used, condensation and debris in the surgery port during surgery may result in dirt on laparoscope lens, leading to poor visualization of the operative field [3]. Therefore, the laparoscopic ports are often cleaned in various ways before insertion of the laparoscope in clinical practice [3, 4]. The surgical gauze grasped with surgical forceps or laparoscopic forceps has customarily been used to clean the port. However, the gauze method is not optimized for the port cleaning and has problems with the risk of tearing loose gauze fragments, breaking port valves, and incurring intra-abdominal organ injury by forceps. [5]

A quick, efficient, and standardized cleaning of the laparoscopic port prior to insertion of the laparoscope will lead to shorter operative time and better procedure of surgery. Therefore, we have developed two different dedicated cleaning devices for laparoscopic port cleaning: a cylinder-type cleaner (Endo Wiper; Osaki Medical Co. Ltd, Aichi, Japan) and a swab-type cleaner (Port Cleaner; Hakuzo Medical, Osaka, Japan). These dedicated devices or gauze cleaning methods are currently used in each hospital, depending on the surgical techniques and surgeon preference, but no comparison has ever been made and each feature is not clearly defined. In addition, there are few reports of different port cleaning techniques despite the fact that they are an important intraoperative procedure, and no standardized method is currently established.

In this study, we have compared the performance of different cleaning techniques with or without using dedicated devices with the addition of our developed swab-type cleaner by a new quantitative method using pseudo-blood and absorbance spectrophotometry. The detailed comparison in cleaning performance was also conducted between the two dedicated devices, using the latest industrial evaluation technology.

## Materials and methods

### Port cleaning methods

**Fig. 1:**
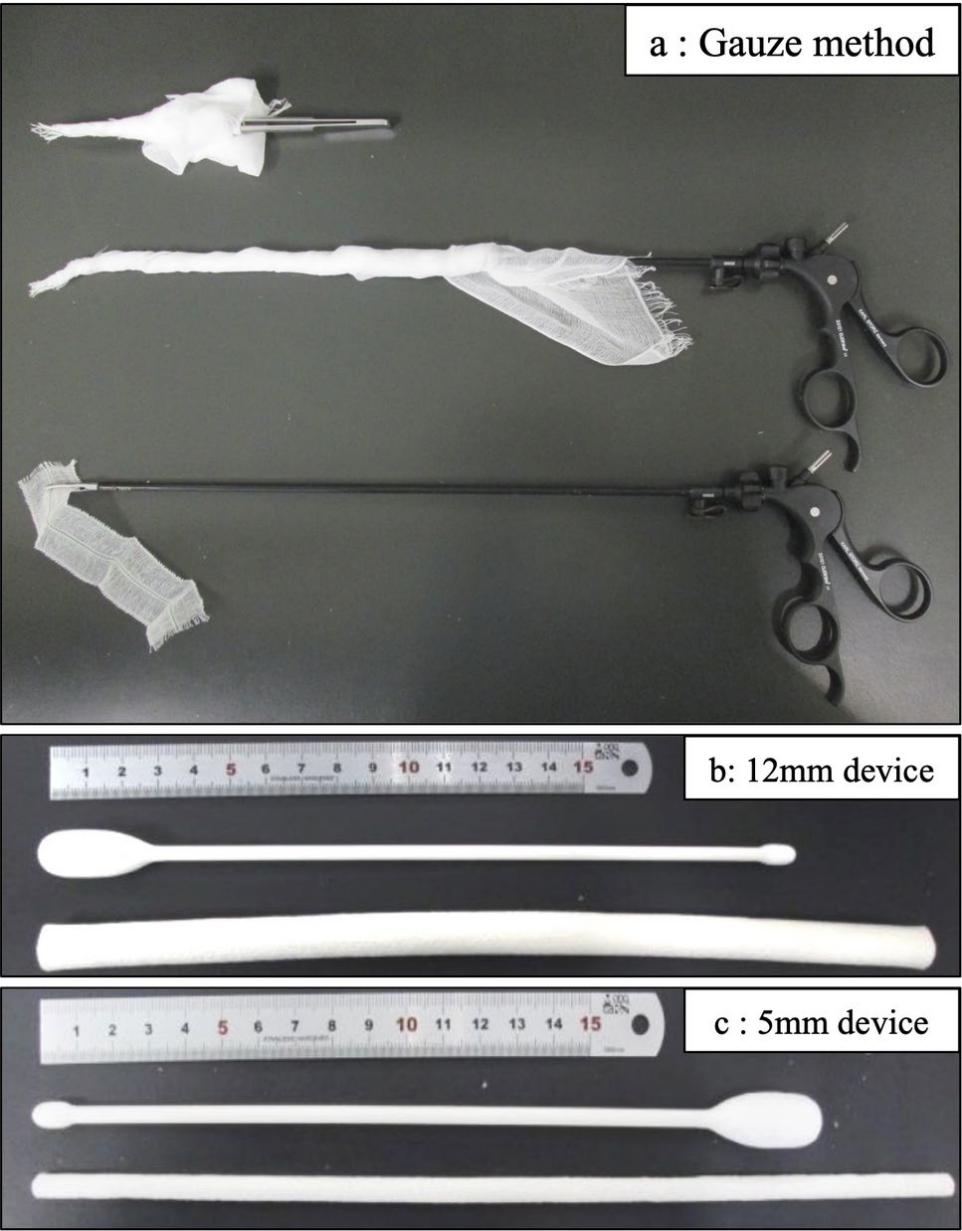
Port cleaning methods. **a** Three cleaning methods with surgical gauze. **b** Dedicated cleaning devices for 12-mm port. **c** Dedicated cleaning devices for 5-mm port

Three previously reported port cleaning methods with surgical gauze already were selected in this study. The first method uses a 30 × 30 cm large surgical gauze (sterilized opegauze G; Hakuzo Medical, Osaka, Japan) wrapped around the 180-mm surgical forceps, and the second method is a 30 × 30 cm large surgical gauze wrapped around the laparoscopic forceps (CLICK line, K33310CC; Karl Storz, Tuttlingen, Germany), and the third method uses a 3 × 15 cm small laparoscopic gauze (laparogauze G; Hakuzo Medical, Osaka, Japan) wrapped around the laparoscopic forceps (Fig. 1a). Two dedicated cleaning devices, a cylinder-type cleaner and a swab-type cleaner, were used (Fig. 1b, c).

The standard 12-mm and 5-mm laparoscopic ports (Versa One; Covidien) were used in this study. To simulate the surgical environment, we used pseudo-blood (Mock Blood Venous; Limbs&Things, Bristol, UK) to smudge the port as reported in the past [5]. The pseudo-blood was injected into the tip of the port sleeve using 0.2 ml for the 12-mm port and 0.08 ml for the 5-mm port, and then it was turned sideways. The port was then tilted and pseudo-blood was extended to the sleeve, 10 cm from the port tip for the 12-mm port, and 7 cm for the 5-mm port. Finally, the port was rotated 2.5 times to create uniform smudge. The cleaning was then attempted by passing the gauze/forceps through and out of the port three times.

### Quantification of port cleanings test using UV spectrophotometer

We quantified the amount of pseudo-blood remaining in the port after cleaning by measuring the absorbance of the pseudo-blood using a UV spectrophotometer (V-630UV spectrophotometer; JASCO, Tokyo, Japan). First, the absorbance of adhering to the ports was measured before cleaning as a control. The ports with adherent pseudo-blood were immersed in pure water (12-mm port: 50 mL pure water, 5-mm port: 20 mL pure water) and the absorbance of this solution was measured (500 nm). This procedure was repeated five times and the median value was defined as the control for the absorbance of the pseudo-blood on a pre-cleaning port. The same procedure was performed after the above five different methods of port cleaning and the absorbance was measured. Finally, the ‘port cleaning rate’ was calculated using the following formula.

$$Port\;cleaning\;rate\left( \% \right) = \frac{{\left( {the\;absorbance\;before\;cleaning} \right) - \left( {the\;absorbance\;after\;cleaning} \right)}}{{the\;absorbance\;before\;cleaning}} \times 100$$

At each typical port cleaning rate after port cleaning (95%, 90%, 80%, 60%), how the laparoscopic image would look clinically was evaluated when the laparoscope was actually used in live swine models under general anesthesia.

### Comparison of port cleaning rates by single use

To compare single-use port cleaning rates, all five methods described above were performed five times each for cleaning the 12-mm port. For cleaning the 5-mm port, a 30 × 30 cm large surgical gauze could not be used, so only three other methods (small laparoscopic gauze + laparoscopic forceps, a cylinder-type cleaner, and a swab-type cleaner) were used five times each.

### Comparison of port cleaning durability by multiple use

The durability of the dedicated cleaning devices was assessed after continuous use. The port cleaning rates were calculated after the first, third, and fifth use of the same device, respectively. This series of procedures was performed five times and the degree of reduction in the cleaning rate was compared between the two devices.

### Assessment for the contact of dedicated cleaning devices with port sleeves during cleaning

**Fig. 2:**
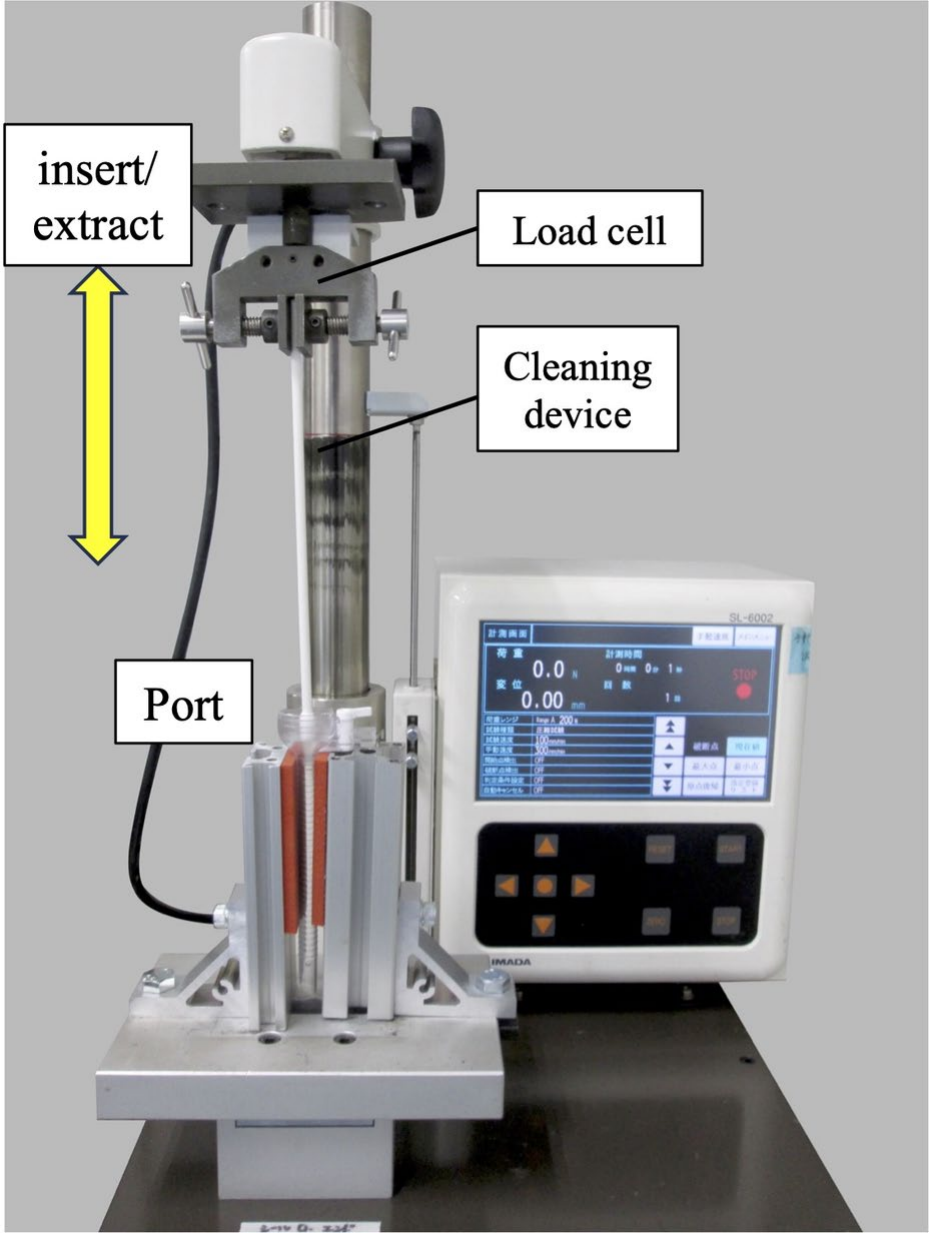
The method of contact load test. The port was set in the fixture and the cleaning device was attached to the load cell straight on the movable axis. The tension and compression testing machine (SVZ-50NB-20R1; Imada Corporation, Aichi, Japan) was moved at a constant speed (100 mm/min), and the maximum contact load (N) when the device was inserted/extracted was measured

Contact load tests and the microfocus CT were performed to confirm the contact between the two dedicated devices and the port sleeve during cleaning procedures. The contact load was measured using a tension and compression testing machine. (SVZ-50NB-20R1; Imada Corporation, Aichi, Japan) (Fig. 2). The port was set in the fixture and the cleaning device was attached to the load cell straight on the movable axis. The port valve was removed, since this study was to measure the contact load between the device and the port sleeve. The tension and compression testing machine was moved at a constant speed (100 mm/min), and the maximum contact load (N) when the device was inserted/extracted was measured five times.

Non-destructive CT imaging was also performed using MCT225 micro-CT scanner (NIKON SOLUTIONS CO., LTD., Tokyo, Japan) to visualize the contact area when the device was inserted into the port.

### Water absorption measurement of dedicated cleaning devices

To assess the blood absorption capacity of the two dedicated cleaning devices, water absorption measurements were performed on each device. The water absorption was measured only on the part of cotton swab for a swab-type cleaner. For a cylinder-type cleaner, it was measured on the identical portion to the cotton swab portion (3.3 cm for the 12-mm port and 1.2 cm for the 5-mm port). The water absorption was simply calculated as the increase in weight when the cleaning part of the device was immersed in water.

### Statistical analysis

Statistical analyses were performed using a dedicated statistical software package (JMP version 17.0.0; SAS Institute, Cary, NC, USA) on a universal personal computer. Data were given as the mean ± standard error (SE). Statistical differences for comparison of port cleaning rates by single use, the contact loads, and water absorption were calculated by using the t-test. Comparison of port cleaning durability by multiple use between one time and five times was analyzed using paired t-test. A p-value of < 0.05 was considered statistically significant.

## Results

### Port cleaning rate

**Fig. 3:**
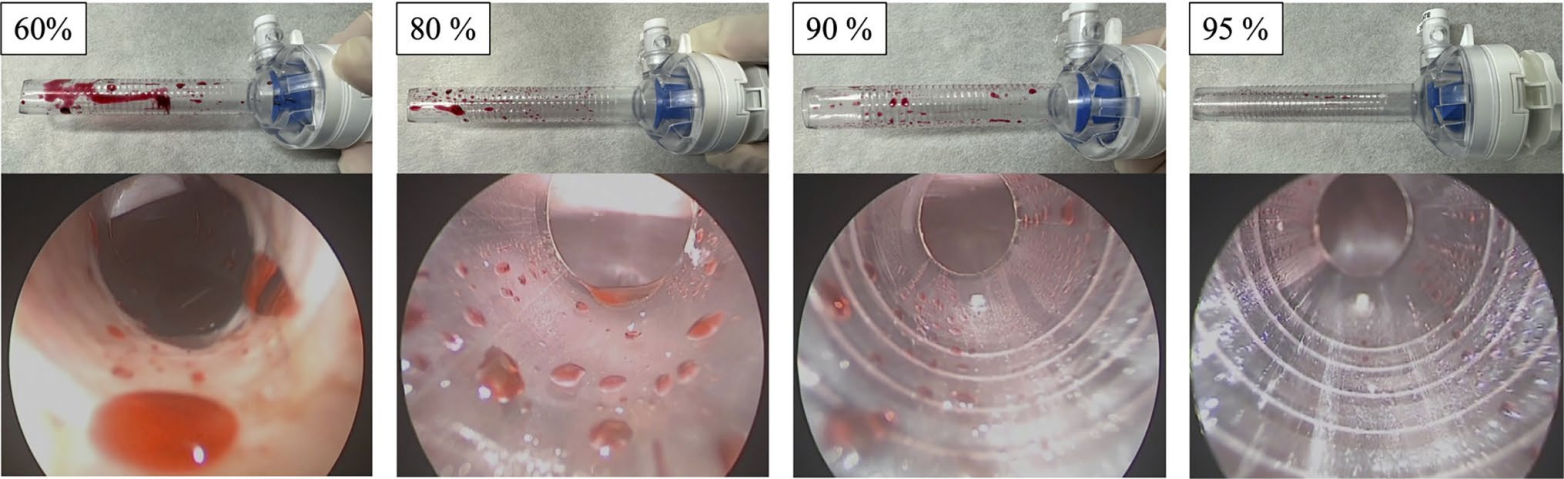
The laparoscopic image for each typical port cleaning rate after port cleaning. If the port was cleaned with values ranging from 60 to 95%, the clinical laparoscopic image showed how much pseudo-blood remained when laparoscope was inserted into the port

The absorbance of the pseudo-blood on a pre-cleaning port is shown in Supplementary Table 1, with a median value of 0.286 for the 12-mm port and 0.300 for the 5-mm port. These values were used as the control for calculating the ‘port cleaning rate.’ Fig. 3 shows the laparoscopic image for each typical port cleaning rate after cleaning procedure (95%, 90%, 80%, 60%) with porcine model. If the port was cleaned with representative values ranging from 60 to 95%, the clinical laparoscopic image showed how much pseudo-blood remained when the laparoscope was inserted into the port after cleaning.

### Comparison of port cleaning rates by single use

**Fig. 4:**
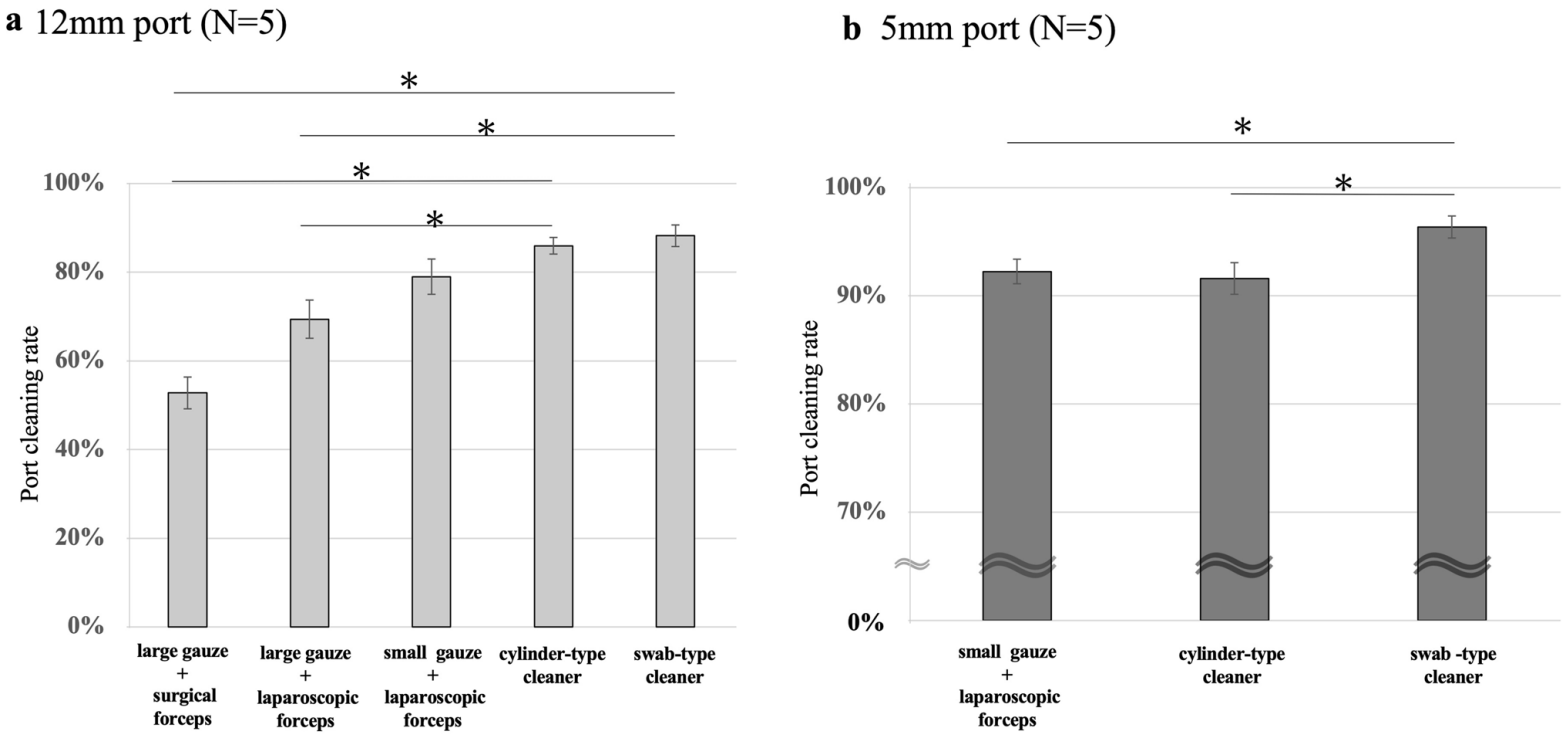
Comparison of port cleaning rates by single use. **a** The port cleaning rate of each of the five cleaning methods for the 12-mm port. **b** The port cleaning rate for the 5-mm port. Data represent mean ± standard error (*n* = 5), *p < 0.05

The mean port cleaning rates of each of the five cleaning methods for the 12-mm port are shown in Fig. 4a; large gauze + surgical forceps was 52.8%, large gauze + laparoscopic forceps was 69.4%, small gauze + laparoscopic forceps was 79.0%, a cylinder-type cleaner was 86.0%, and a swab-type cleaner was 88.2%, respectively. Both port cleaning rates of the two devices were significantly higher than the methods using large surgical gauze (*p* < 0.05 for both). In a comparison of the two cleaning devices and small gauze + laparoscopic forceps, the mean port cleaning rate was higher for the two cleaning devices, but both were not significantly different (small gauze vs. a cylinder-type cleaner, *p* = 0.144; small gauze vs. a swab-type cleaner, *p* = 0.095). The mean port cleaning rates of each of the three cleaning methods for the 5-mm port are shown in Fig. 4b; small gauze + laparoscopic forceps was 92.3%, a cylinder-type cleaner was 91.6%, and a swab-type cleaner was 96.4%, respectively. A high cleaning rate was observed in all three methods, and a swab-type cleaner showed the highest cleaning rate compared to small gauze + laparoscopic forceps and a cylinder-type cleaner (small gauze vs. a swab-type cleaner, *p* = 0.028; a cylinder-type cleaner vs. a swab-type cleaner, *p* = 0.032).

### Comparison of port cleaning durability by multiple use

**Fig. 5:**
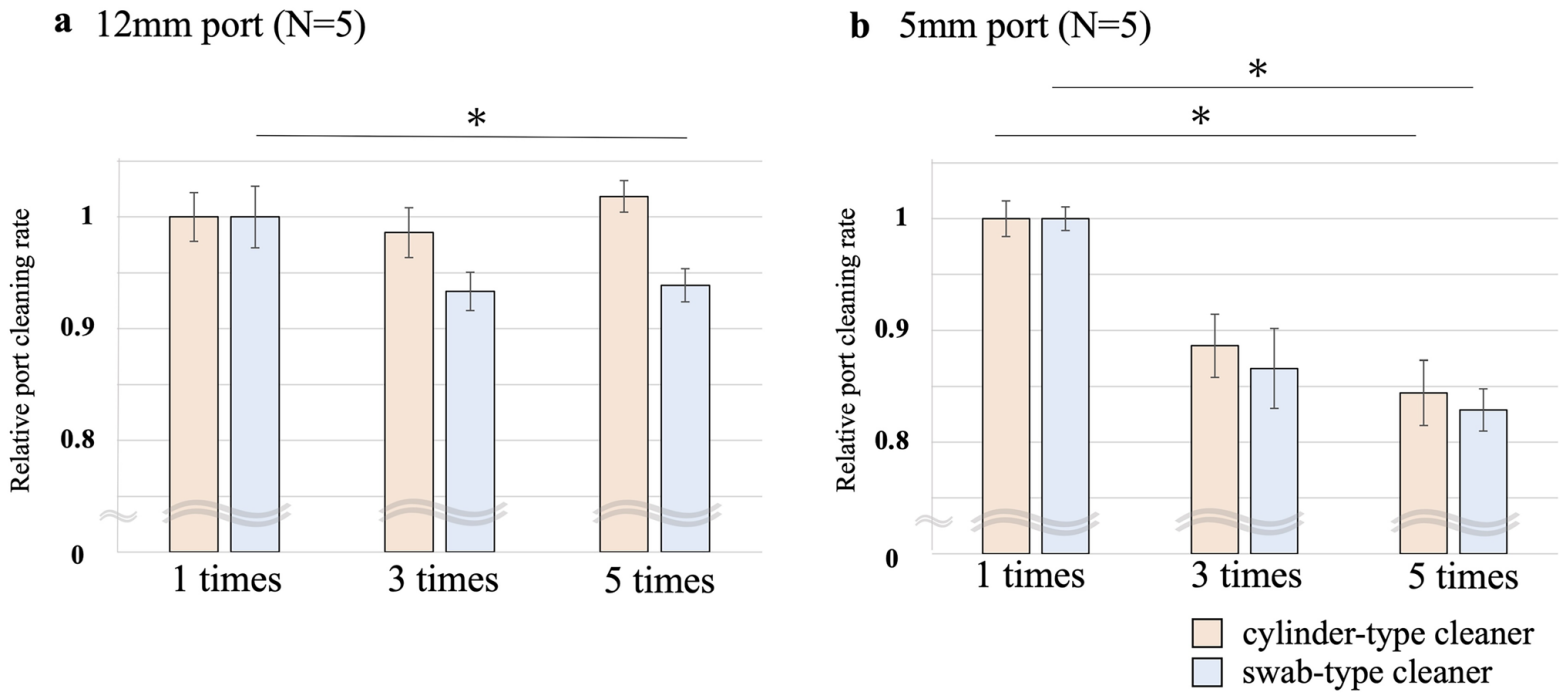
Comparison of port cleaning durability by multiple use. The change in port cleaning rates of the two dedicated cleaning devices with continuous cleaning for the 12-mm port (**a**) and the 5-mm port (**b**). The first cleaning rate is shown as the control, and the third and fifth cleaning rates are shown as relative bars. *p < 0.05

The change in port cleaning rates of the two dedicated cleaning devices with continuous cleaning for the 12-mm port is shown in Fig. 5a. A swab-type cleaner significantly reduced the cleaning rate by an average of 5.4% after five consecutive cleanings (1 time vs. 5 times; *p* = 0.044). On the other hand, a cylinder-type cleaner showed no reduction in cleaning rate after five consecutive cleanings. The change in port cleaning rates for the 5-mm port is then shown in Fig. 5b. Consecutive cleaning of the 5-mm port significantly reduced the cleaning rate by an average of 14.3% for cylinder-type cleaner (*p* = 0.003) and 16.5% for a swab-type cleaner (*p* = 0.004) compared to the 1 time and 5 times cleaning.

### Assessment for the contact of dedicated cleaning devices with port sleeves during cleaning

**Fig. 6:**
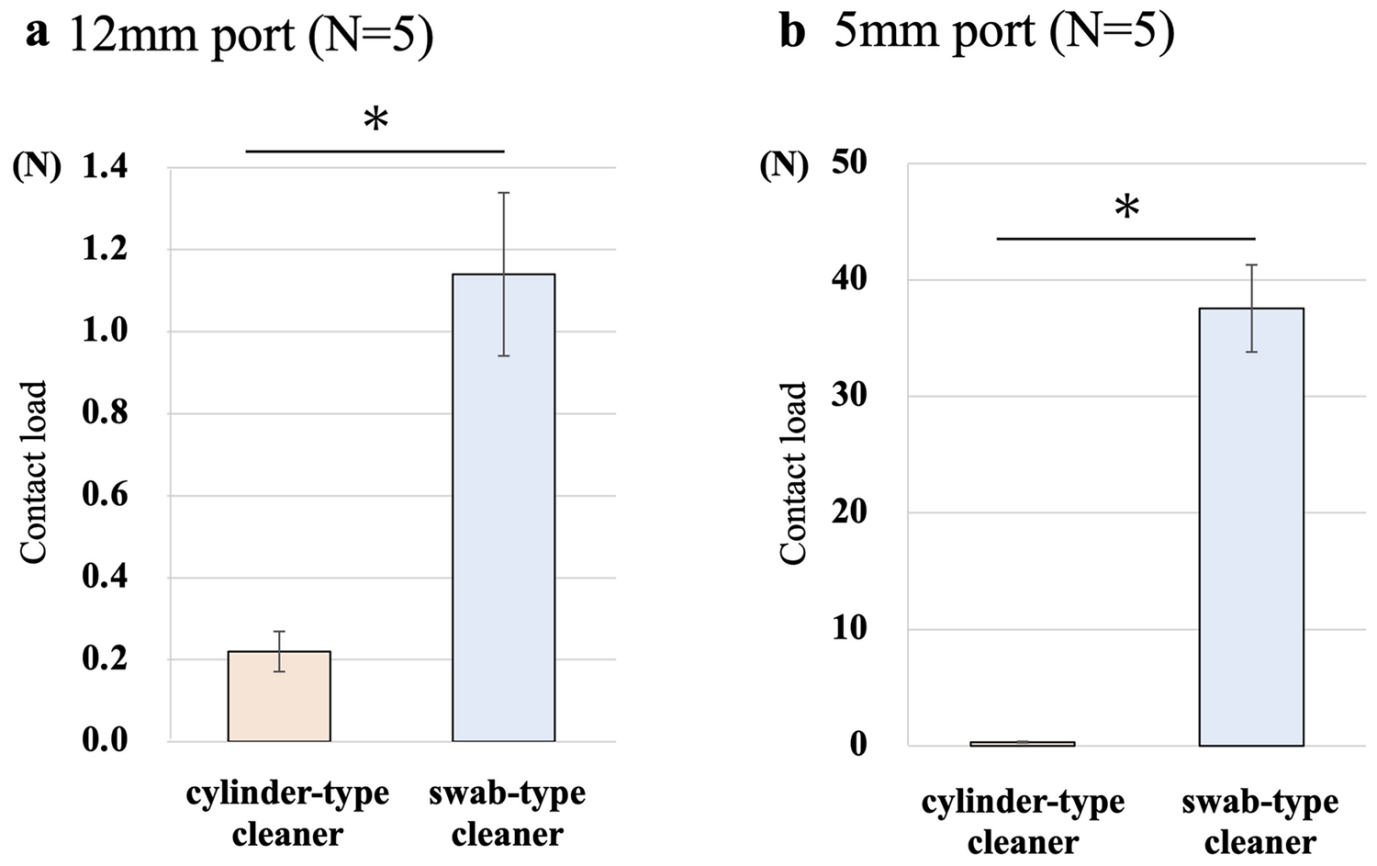
The contact load test. The contact load test between cleaning devices and the port sleeve (**a**: 12-mm port, **b**: 5-mm port) using a tension and compression testing machine. Data represent mean ± standard error (*n* = 5), *p < 0.05

**Fig. 7:**
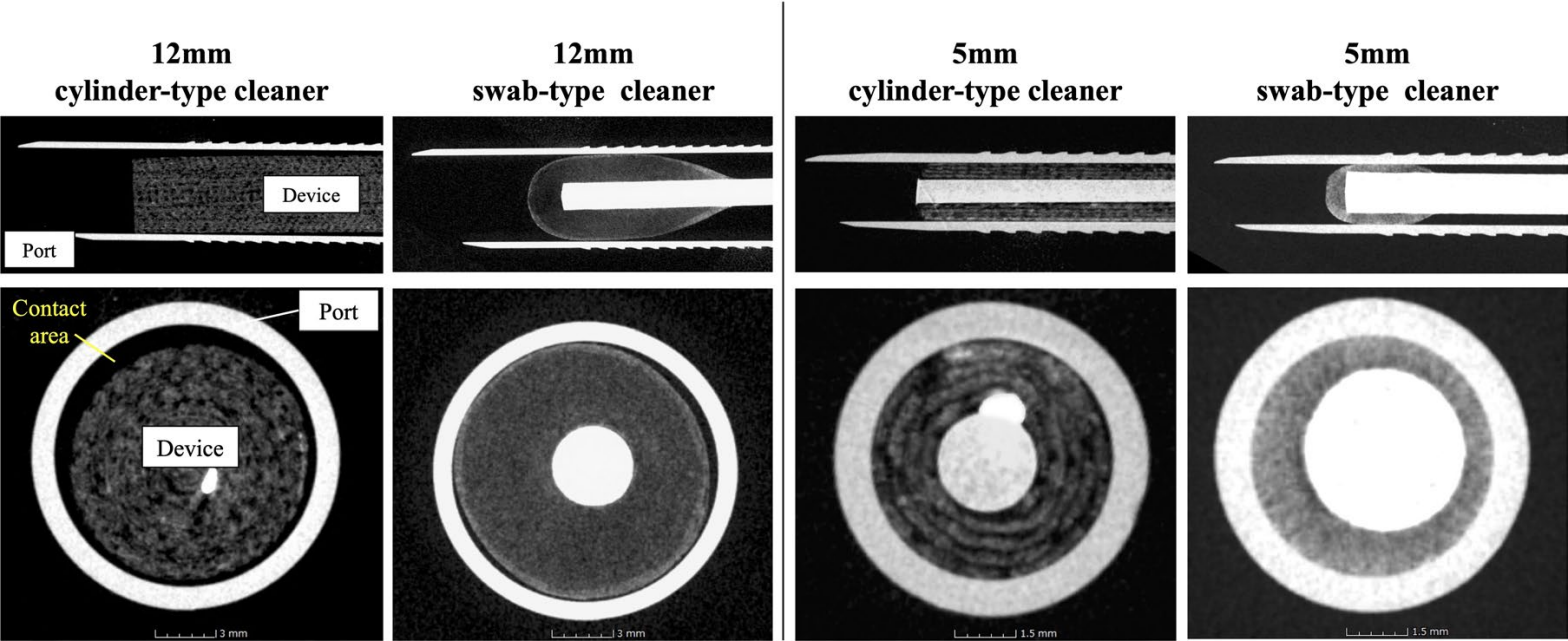
Visualization for contact area of dedicated cleaning devices. Sagittal section and axial section of microfocus CT scans with each device inserted into the port are shown. The contact area (yellow text) is the space between the device and the port sleeve (Color figure online)

Figure 6 shows the results of the contact load test between cleaning devices and the port sleeve using a tension and compression testing machine. In the 12-mm port, the maximum contact load with a cylinder-type cleaner was on average 0.22 N and with a swab-type cleaner was on average 1.14 N, resulting in a significantly higher contact load with a swab-type cleaner (*p* = 0.008). Similarly, in the 5-mm port, the maximum contact load with a cylinder-type cleaner was on average 0.32 N and with a swab-type cleaner was on average 37.56 N, and the contact load was statistically higher with a swab-type cleaner (*p* < 0.001). Sagittal section and axial section of microfocus CT scans with each device inserted into the port are shown in Fig. 7. We visually demonstrated that a swab-type cleaner had a larger contact area for both 12-mm and 5-mm devices than a cylinder-type cleaner.

### Water absorption measurement of dedicated cleaning devices

**Fig. 8:**
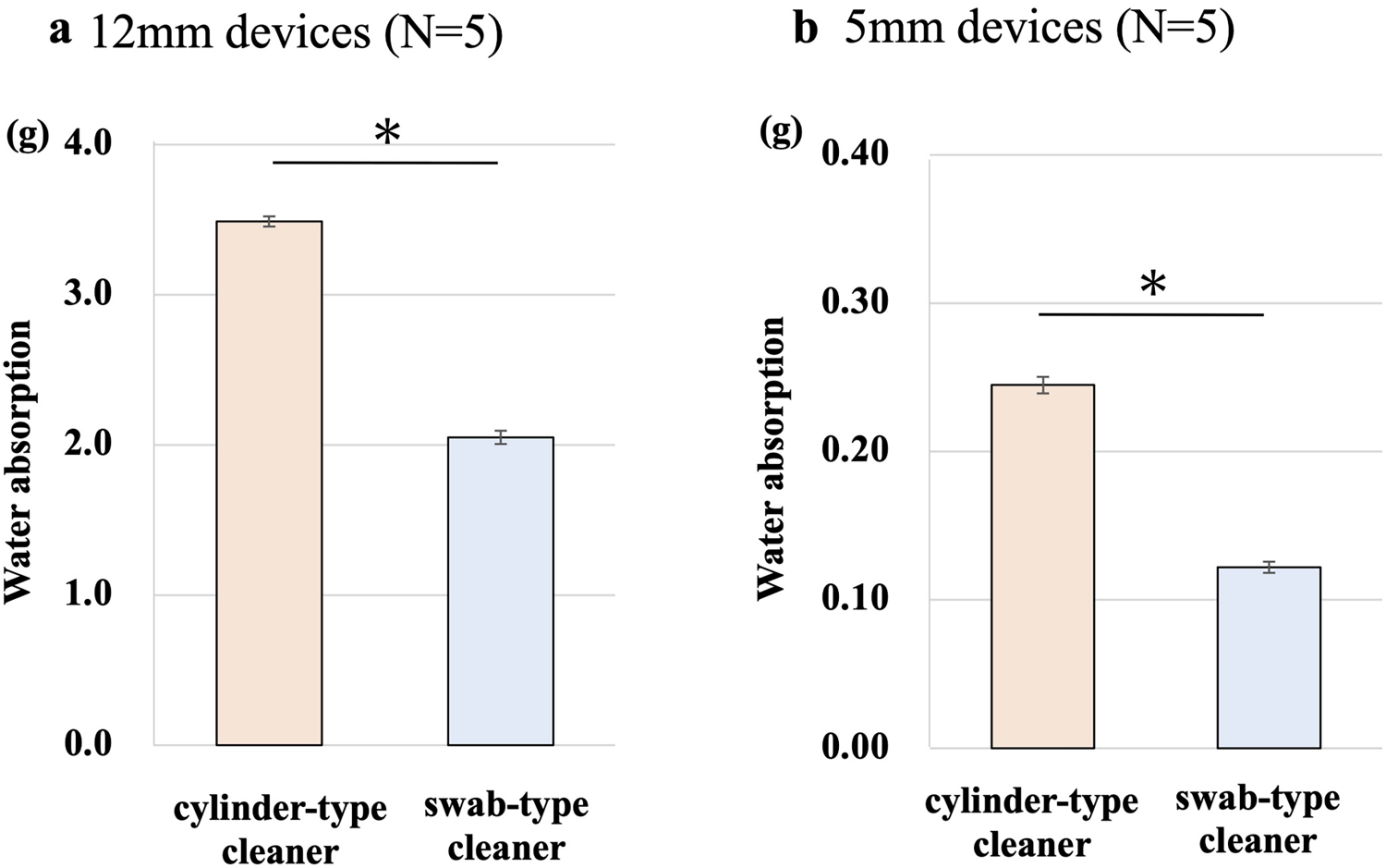
Water absorption measurement. The results of water absorption measurement are shown (**a**: 12-mm devices, **b**: 5-mm devices). Data represent mean ± standard error (*n* = 5), *p < 0.05

In 12-mm dedicated cleaning devices, a cylinder-type cleaner absorbed an average 3.49 g of water and a swab-type cleaner absorbed an average of 2.05 g. In 5-mm dedicated cleaning devices, a cylinder-type cleaner absorbed an average 0.24 g of water and a swab-type cleaner absorbed an average of 0.12 g. Water absorption of a cylinder-type cleaner was significantly higher than a swab-type cleaner in both 12-mm and 5-mm devices (12 mm, *p* < 0.001; 5 mm, *p* < 0.001; Fig. 8).

## Discussion

Recent advances in quality of images in laparoscopic surgery, such as 3D and 4 K technologies, have enabled more detailed anatomical recognition and surgical manipulation, leading to improved surgical outcome [1, 2, 6, 7]. This has increased the demand for cleanliness of the surgical visualization, and there have been many reports on the development of dedicated devices for cleaning the lens of the laparoscopes [8–11]. However, there are few comparative studies on the cleaning for the laparoscopic port, although efficient port cleaning to remove water droplets and blood before laparoscope reinsertion is also important to keep the lens clean. Many surgeons still perform port cleaning using customary their own methods, such as cleaning with gauze and forceps and laparoscopic forceps. We have therefore started developing a dedicated port cleaning device since 2016 with the aim of enabling port cleaning to be performed more efficiently and quickly in a more standardized fashion [5].

The conventional gauze methods have problems with the gauze end migrating into the abdominal cavity, air leakage from the valve due to damaged laparoscopic valves, and the risk of injury to intra-abdominal organs by forceps [5]. In addition, the cleaning method using gauze is not optimized for the port diameter, so the gauze may not reach the tip of the port sleeve, resulting in inadequate cleaning. Our present study quantitatively demonstrated that the two dedicated devices were superior to the conventional gauze methods for cleaning both 12-mm and 5-mm ports, suggesting that dedicated devices should be actively used for cleaning laparoscopic ports.

In a comparison of the cleaning performance of a cylinder-type and a swab-type cleaner, a swab-type cleaner had better port cleaning rate in single use, especially for the 5-mm port. With regard to the difference in single cleaning rates, we consider that the contact area between the device and the port sleeve has a significant influence. Therefore, in this study, this contact area was assessed quantitatively by contact load test and visually by microfocus CT. Both contact load and contact area were higher for a swab-type cleaner in 12-mm and 5-mm ports. A swab-type cleaner was designed with a larger diameter as a product, which we believe has led to the higher contact load and contact area in this study. On the other hand, prioritizing the cleaning rate by increasing the diameter of devices can lead to difficulty in insertion and extraction, resulted in a poor user experience. Thus, in the development of dedicated port cleaning devices, the balance between cleanability and ease of device traffic is considered important.

There are differences in the feature of the two dedicated cleaning devices and how they are used during surgery. A swab-type cleaner can clean both the 5-mm and 12-mm ports with one swab. It is very useful in operations when a 5-mm laparoscope is used, as the laparoscope may be inserted through both the 5-mm and 12-mm ports to change the field of view. Even if only the 12-mm port is used as a scope port, a swab-type cleaner has advantages, e.g., the remaining 5-mm swab can be used to clean the laparoscope lens. In addition, it is also possible to reduce the number of surgical items to be managed in operating rooms. A cylinder-type cleaner needs to be used separately for 12-mm and 5-mm ports according to the surgical port setting, but its durability was significantly better than that of a swab-type, especially for cleaning the 12-mm port. This is due to the fact that all parts of the product have a uniform cylindrical shape that can be cleaned and it has demonstrated higher water absorbency than a swab-type cleaner. As for the costs, a swab-type cleaner costs $3.2 per one swab and a cylinder-type cleaner costs $5.5 on the Japanese market in 2024, respectively. Although the number of units used during surgery varies depending on the type of procedure and duration of the operation, an understanding of these features and costs of cleaning devices should assist in device selection.

We agree that this study has several limitations. First, our study was experimental using pseudo-blood, and it was not tested on human body. Second, the gauze method in this study was tested by standardizing the way gauze was wrapped around forceps and the cleaning method, but there are various other ways in actual clinical practice. Finally, we use only one commercially available laparoscopic port, but it has not been experimented with ports made by other companies.

## Conclusions

In cleaning laparoscopic ports, dedicated cleaning devices are significantly superior to conventional gauze cleaning methods, and the active use of dedicated devices could be considered. The study also revealed the features of a swab-type cleaner and a cylinder-type cleaner. The advantages and features of these dedicated devices should be well understood, and the cleaning method should be selected according to the environment and surgical techniques.

## Supplementary Information

Below is the link to the electronic supplementary material.

Supplementary file1 (DOCX 14 KB)
