# Supplementary material for: Role of dedicated port cleaning devices in laparoscopic surgery
Source: Surg Endosc. 2024 Nov 11;38(12):7613–20. doi: 10.1007/s00464-024-11366-w (PMC11615053; doi:10.1007/s00464-024-11366-w)
Supplement: Supplementary file 1 — Supplementary file1 (DOCX 14 KB) [file 464_2024_11366_MOESM1_ESM.docx]

**Supplementary Table 1**

The absorbance of the pseudo-blood on a pre-cleaning port

|  | N1 | N2 | N3 | N4 | N5 | median |
| --- | --- | --- | --- | --- | --- | --- |
| 12 mm port | 0.284 | 0.290 | 0.287 | 0.286 | 0.284 | 0.286 |
| 5 mm port | 0.312 | 0.297 | 0.300 | 0.302 | 0.295 | 0.300 |
